# Clinical and cost effectiveness of pulmonary rehabilitation for people with post-tuberculosis lung disease in Kyrgyzstan: a single-blind randomized controlled trial

**DOI:** 10.1093/annalsats/aaoag062

**Published:** 2026-03-23

**Authors:** Azamat Akylbekov, Mark W Orme, Jesse A Matheson, Matthew Richardson, Aijan Taalaibekova, Maamed Mademilov, Gulzada Mirzalieva, Kamila Magdieva, Aigul Ozonova, Aidai Erkinbaeva, Syimyk Azizbekov, Nurdin Shakiev, Uulzhan Bekbolsunova, Aichurok Alymbekova, Kaldygul Dushimbekova, Andy Barton, Michael C Steiner, Dominic Malcolm, Talant Sooronbaev, Sally J Singh

**Affiliations:** Deaprtment of Pulmonology, National Centre of Cardiology and Internal Medicine named after academician M. Mirrakhimov, Bishkek, Kyrgyzstan; Department of Respiratory Sciences, University of Leicester, Leicester, United Kingdom; Centre for Exercise and Rehabilitation Science, National Institute for Health and Care Research Leicester Biomedical Research Centre–Respiratory, University Hospitals of Leicester NHS Trust, Leicester, United Kingdom; Department of Economics, University of Sheffield, Sheffield, United Kingdom; Department of Respiratory Sciences, University of Leicester, Leicester, United Kingdom; Deaprtment of Pulmonology, National Centre of Cardiology and Internal Medicine named after academician M. Mirrakhimov, Bishkek, Kyrgyzstan; Deaprtment of Pulmonology, National Centre of Cardiology and Internal Medicine named after academician M. Mirrakhimov, Bishkek, Kyrgyzstan; Deaprtment of Pulmonology, National Centre of Cardiology and Internal Medicine named after academician M. Mirrakhimov, Bishkek, Kyrgyzstan; Deaprtment of Pulmonology, National Centre of Cardiology and Internal Medicine named after academician M. Mirrakhimov, Bishkek, Kyrgyzstan; Deaprtment of Pulmonology, National Centre of Cardiology and Internal Medicine named after academician M. Mirrakhimov, Bishkek, Kyrgyzstan; Deaprtment of Pulmonology, National Centre of Cardiology and Internal Medicine named after academician M. Mirrakhimov, Bishkek, Kyrgyzstan; Deaprtment of Pulmonology, National Centre of Cardiology and Internal Medicine named after academician M. Mirrakhimov, Bishkek, Kyrgyzstan; Deaprtment of Pulmonology, National Centre of Cardiology and Internal Medicine named after academician M. Mirrakhimov, Bishkek, Kyrgyzstan; Deaprtment of Pulmonology, National Centre of Cardiology and Internal Medicine named after academician M. Mirrakhimov, Bishkek, Kyrgyzstan; Deaprtment of Pulmonology, National Centre of Cardiology and Internal Medicine named after academician M. Mirrakhimov, Bishkek, Kyrgyzstan; Department of Phthisiopulmonology, Kyrgyz State Medical Institute of Post-Graduate Training and Continuous Education named after S. B. Daniyarov, Bishkek, Kyrgyzstan; Department of Respiratory Sciences, University of Leicester, Leicester, United Kingdom; Department of Respiratory Sciences, University of Leicester, Leicester, United Kingdom; Centre for Exercise and Rehabilitation Science, National Institute for Health and Care Research Leicester Biomedical Research Centre–Respiratory, University Hospitals of Leicester NHS Trust, Leicester, United Kingdom; School of Sport, Exercise and Health Sciences, Loughborough University, Loughborough, United Kingdom; Deaprtment of Pulmonology, National Centre of Cardiology and Internal Medicine named after academician M. Mirrakhimov, Bishkek, Kyrgyzstan; Department of Respiratory Sciences, University of Leicester, Leicester, United Kingdom; Centre for Exercise and Rehabilitation Science, National Institute for Health and Care Research Leicester Biomedical Research Centre–Respiratory, University Hospitals of Leicester NHS Trust, Leicester, United Kingdom

**Keywords:** pulmonary rehabilitation, post-TB lung disease, tuberculosis, chronic respiratory disease, exercise

## Abstract

**Rationale:**

Tuberculosis (TB) is a major worldwide cause of disability, with TB survivors experiencing a significant and often underrecognized burden, and approximately half going on to develop post-tuberculosis lung disease (PTLD). Pulmonary rehabilitation may offer effective disease management, but there is a lack of evidence in PTLD populations.

**Objectives:**

We aimed to determine the clinical and cost effectiveness of pulmonary rehabilitation for adults living with PTLD in Kyrgyzstan.

**Methods:**

A single-blind randomized controlled trial, conducted March 2021 to June 2022 in Bishkek, Kyrgyzstan, compared supervised pulmonary rehabilitation to usual care for adults living with PTLD. Participants were randomized (1:1) to receive either usual care (control) or culturally adapted pulmonary rehabilitation (intervention), comprising individually prescribed and tailored exercise and self-management education. The primary outcome was change in maximal exercise capacity, measured by the incremental shuttle walking test (ISWT), from baseline to the end of 6 weeks of pulmonary rehabilitation, analyzed by intention-to-treat analysis. Secondary outcomes included health-related quality of life (HRQoL) and cost-effectiveness analysis.

**Results:**

One hundred fourteen participants (mean ± SD, 43.3 ± 15.2 years; 57% male) received either supervised pulmonary rehabilitation or usual care. Compared with the control group, changes in exercise capacity and HRQoL from baseline were significantly greater in the intervention group (ISWT: 123.0 m [95% CI, 81.2-164.8 m], *P* <.001; EQ-5D-5L Visual Analogue Scale: 20.2 [95% CI 15.5-24.9], *P* <.0001). The intervention group saw a significant increase in quality-adjusted life-years (QALYs) over the control group (0.2 [95% CI, 0.1-0.2]). We calculated a total program cost of US$5686.5 (US$95 per patient who received pulmonary rehabilitation), giving a program cost, after adjusting for purchasing power, of US$2143.2 per QALY (95% CI, $1621.9-$2663.9).

**Conclusions:**

In adults with PTLD in Kyrgyzstan, a culturally adapted pulmonary rehabilitation program significantly improved exercise capacity and HRQoL compared with usual care and was both clinically and cost effective.

## Introduction

Tuberculosis (TB) is a major worldwide cause of disability and death, with >10 million new cases annually^1^^,^^2^ and approximately 1.3 million deaths in 2022.^3^ In 2020, there were an estimated 155 million TB survivors globally,^4^ with an estimated 70% receiving treatment.^3^ The burden of TB falls predominantly in low- and middle-income countries (LMICs).^5^^,^^6^ Kyrgyzstan is a lower-middle-income country in Central Asia with a high poverty level (20.1%),^7^^,^^8^ the highest incidence rate of TB within the World Health Organization (WHO) European Region (130 per 100 000),^9^ and one of the highest respiratory-related mortality rates (19.5 per 100 000).^10^^,^^11^

TB survivors continue to experience a significant and often underrecognized burden, even after completing treatment,^12-15^ with approximately half of survivors going on to develop post-tuberculosis lung disease (PTLD), a form of chronic respiratory disease (CRD).^16-18^ PTLD has not traditionally been a major focus of the TB control agenda, but the needs of people living with PTLD are becoming increasingly recognized.^19^ PTLD results in structural abnormalities and anatomical distortion, along with abnormal respiratory physiology characterized by modified lung volumes and compromised diffusing capacity.^20^ In Kyrgyzstan, as in many post-Soviet countries, TB specialists commonly use the terms “minor residual changes after a special process (tuberculosis) in the lungs” and “major residual changes after a special process in the lungs” to describe post-TB radiological findings. These classifications are based on chest X-ray interpretation and reflect the extent of residual pulmonary damage.^21^ PTLD detrimentally affects quality of life, impairs physical functioning, and causes chronic respiratory symptoms.^22^ The physical effects of PTLD are often accompanied by social isolation and depression,^19^^,^^23-25^ exacerbated by stigmatization of people living with TB even after recovery or treatment completion.^19^^,^^25-27^

With anticipated rises in the number of TB survivors, clinically and cost effective post-treatment interventions are urgently required. While PTLD has been regarded as a substantial challenge in the WHO policy brief on TB-associated disability, post-TB care remains neglected.^28^ The WHO Rehabilitation 2030 initiative advocates increasing the availability of high-quality evidence for rehabilitation. Pulmonary rehabilitation is a comprehensive program that encompasses personalized exercise, education, and self-management activities, targeted at those with a high symptom burden secondary to their CRD. International guidelines recommend pulmonary rehabilitation as a non-pharmacological treatment for various CRDs,^29^ but it is not currently recommended in PTLD largely due to a lack of evidence.^30^ In a study of short-term pulmonary rehabilitation for people living with PTLD during the COVID-19 pandemic, in India, improvements in functional capacity, quality of life, and dyspnea were observed, supporting the potential for pulmonary rehabilitation in a PTLD population.^31^ Given that most people living with PTLD are younger than the majority of those with CRDs, the economic impact within this group is amplified.^4^

A systematic review of pulmonary rehabilitation in LMICs identified 13 studies, of which 11 were at high risk of bias and 2 were moderate risk of bias.^32^ No studies were conducted in Central Asia, and only 1 (pilot) study included participants with PTLD (South Africa, moderate risk of bias). Considering the cultural and contextual differences, Western models of pulmonary rehabilitation may not be suitable for implementation in LMICs, underscoring the importance of adapting the program to the specific context of Kyrgyzstan and PTLD.^31^^,^^33^ Our previous qualitative work revealed a highly positive reception of pulmonary rehabilitation by both patients and healthcare workers and informed our pulmonary rehabilitation intervention.^34^^,^^35^ Accordingly, we conducted a randomized controlled trial to determine the clinical and cost effectiveness of culturally adapted pulmonary rehabilitation in a novel PTLD population. We hypothesized that culturally adapted supervised pulmonary rehabilitation will improve maximal exercise capacity compared to usual care in individuals living with PTLD.

## Methods

### Study design

A single-blind randomized controlled trial was conducted to compare a culturally adapted pulmonary rehabilitation with usual care for people living with PTLD. The study took place at the National Centre of Cardiology and Internal Medicine (NCCIM) in Bishkek, Kyrgyzstan. The study was approved on July 22, 2019 by ethics committees at the NCCIM (reference no. 17) and the University of Leicester, United Kingdom (reference no. 22293). The study design has been described in detail elsewhere.^35^ The cultural adaptations incorporated into pulmonary rehabilitation have been previously reported.^34^ All participants provided written informed consent.

### Participants

Eligible patients were those aged 18 years or older, who had completed TB medical treatment, were confirmed TB negative through Ziehl–Neelsen stain^36^ or GenExpert method,^37^ and who reported a Medical Research Council (MRC) dyspnea grade ≥2. PTLD was diagnosed by TB specialists by chest X-ray and sputum analysis. Patients were excluded if they had comorbidities preventing exercise participation, malignancy, evidence of active TB based on chest X-ray or sputum tests, or an inability or unwillingness to provide informed consent. Healthcare professionals, including TB doctors, family doctors, and pulmonologists, referred potentially eligible patients to the research team at NCCIM. The research team confirmed the eligibility criteria status of each patient before enrolling those who provided written informed consent.

### Randomization and masking

The participants were randomly assigned (1:1) to either pulmonary rehabilitation or usual care. Randomization was conducted using sealedenvelope.com^38^ by a dedicated member of the research team. Participants were unblinded to their allocation. Outcome assessors and the data analyst were blinded to group allocation.

### Intervention

Doctors from family medicine centers, City TB Hospital, City TB Dispensary, the National Hospital, district hospitals, and the NCCIM screened patients for eligibility and referred interested individuals to the pulmonary rehabilitation trial team. Upon confirming eligibility and obtaining written informed consent, participants underwent a baseline assessment at the NCCIM, conducted by staff members trained in the measures. Once the baseline measures were completed, participants were randomly assigned (1:1) to either the supervised pulmonary rehabilitation (intervention) group or the usual care (control) group. Outcomes measures were taken at baseline assessments (week 0), at post assessments (at 6 weeks post-baseline), and at follow-up telephone assessments (at 12 weeks post-baseline) where possible. In cases where participants were unable to attend the assessments in person, questionnaire outcomes were administered via telephone.

#### Supervised pulmonary rehabilitation intervention

The PTLD pulmonary rehabilitation program was held twice weekly for 6 weeks (12 sessions) and a maximum capacity of 5 patients per session. The pulmonary rehabilitation program delivery was led by a trained nurse and supported by a multidisciplinary team of a nurse, doctor, and physiotherapist. Each ­session lasted approximately 2 hours, with the first hour dedicated to the educational components to support effective self-management, covering topics such as the respiratory system, pulmonary TB, nutrition, smoking, exercise, and discussions regarding coping with the stigma of TB. Practical sessions on inhalation therapy and medications were also conducted. The second hour focused on individually prescribed exercise. Aerobic and strength training components followed international guidelines.^29^^,^^39^ Strength exercises included bicep curls, pull-ups, sit-to-stands, and step-ups. Participants were prescribed 3 sets of 8 to 12 repetitions of each exercise. In addition to dumbbells, participants exercised with equipment that could be replicated at home, such as bottles filled with water to their appropriate weight. Aerobic exercise included ground-based walking and static cycling. Walking was individually prescribed at a speed equivalent to 85% maximal oxygen capacity (VO_2_ peak) derived from the incremental shuttle walking test (ISWT). Walking was monitored and target duration progressed/adjusted during the program. Patients were asked to do one additional session of strength training and to walk every day at home. Based on our previous qualitative work,^34^ additional activities were incorporated. These were rhythmic movements to music, dancing the national dance “Kara Jorgo,”^40^ and peer-support talking-based therapy to address stigma of TB and providing participants with an opportunity to share their lived experiences and stories. Rhythmic movements and the Kara Jorgo dance were guided by an instructor, with participants engaging in coordinated movements and dancing together to music.

#### Usual care

Usual care followed local best practice, in the absence of national/international guidelines, which included inhaled therapies and usual prescription medications and an educational booklet providing advice about exercise, healthy diet, smoking cessation, and avoiding biomass smoke. Participants in the control group were offered pulmonary rehabilitation at the conclusion of the trial.

### Outcomes

Outcomes aligned with the minimum dataset for trials of pulmonary rehabilitation in LMICs.^41^ No changes to planned outcome measures were made after the trial commenced.

#### Primary outcome

The primary outcome was change in maximal exercise capacity, measured by the ISWT,^42^ from baseline to the 6-week post assessment. The primary metric of interest is the mean value of the outcome for the pulmonary rehabilitation group compared to the mean value of the outcome for the usual care group, with a minimum clinically important difference (MCID) considered 35 m.^43^

#### Secondary outcomes

Health-related quality of life was assessed using the MRC (1 item) dyspnea grade,^44^ the Clinical COPD Questionnaire (CCQ; 10 items),^45^ and the COPD Assessment Test (CAT; 8 items).^46^ CCQ total score and symptoms and the functional and mental domains are reported, with an MCID of 0.4^47^ for the total score. The MCID was 1 for the MRC^48^^,^^49^ and 2 for the CAT.^50^ The Hospital Anxiety and Depression Scale (HADS; 14 items) was used to assess anxiety and depression, with scores categorized as normal (score 0-7), mild (score 8-10), moderate (score 11-15), or severe (score 16-21)^51^ and an MCID for each domain of 2.^52^^,^^53^ The EuroQol EQ-5D-5L (6 items)^54^ was used to assess quality of life. The EQ-5D-5L comprised domains (mobility, self-care, usual activities, pain/discomfort, anxiety/depression) and Visual Analogue Scale (VAS) ranging from 0 (worst health) to 100 (best health). The MCID for the VAS was 7.^55^ Physical function was assessed using the 5-times sit-to-stand (5STS),^56^ with an MCID of 1.7 seconds.^57^ Exercise endurance was examined by the endurance shuttle walk test (ESWT),^58^ with an MCID of 147 to 279 seconds.^59^

Attendance to pulmonary rehabilitation sessions and the number of additional scheduled sessions were used to describe intervention adherence. Adverse events were recorded, including seriousness, duration, relatedness, severity, action taken, outcome, and treatment arm.

### Health economics

Outcomes for patient quality of life were based on the EQ-5D-5L questionnaire.^60^ The outcome for each of the 5 dimensions was a numerical response (0 to 5), where a higher value indicates a worse outcome. Country-specific value sets for the EQ-5D-5L surveys are used to map EQ-5D-5L survey responses to quality-adjusted life-years (QALYs)^60^^,^^61^ for each observation in the data. No crosswalk values from the EQ-5D-5L to an index value exist for Kyrgyzstan. Instead, crosswalk values for all available countries were used, with main results using the most conservative estimates (corresponding to index values for the country of Zimbabwe).

The change in mean QALY value, from baseline to the 6-week post assessment, was compared between the pulmonary rehabilitation intervention group and the usual care group. This value was combined with program cost estimates to provide a cost per QALY. Costs were adjusted for purchasing power differences between Kyrgyzstan and the United States to ensure comparability between program cost and established cost-per-QALY thresholds. Purchasing power parity adjustment was based on adjustment used by the International Monetary Fund for 2024 Kyrgyz Republic gross domestic product (GDP) adjustments. The factor for adjusting GDP in US$ to GDP at purchasing power parity was 3.49.^62^

### Statistical analysis

The trial was powered to detect a 35-m difference in the primary outcome (ISWT distance),^43^ with an SD taken from a previous feasibility study assessing pulmonary rehabilitation in a PTLD population in Uganda.^63^ The study was powered at 80% with a type I error of 0.05, requiring a trial sample size of 40 participants in each group. Accounting for a 30% loss to follow-up, this gave a target sample size of 114 participants.

All analyses were performed using R software version 4.3.1^64^ and used the ALICE High Performance Computing facility at the University of Leicester. The primary statistical analyses were an intention to treat (ITT) where the population consists of all randomized participants into the trial regardless of whether they received the intervention. A per protocol analysis was performed on all individuals who had complete data on the primary outcome (attending a baseline and discharge assessment) and who adhered to the intervention, defined as attending 75% of face-to-face sessions (≥9 of 12 sessions).^29^

A generalized linear mixed model (GLMM) framework was used to model the repeated measures for each of the outcomes. The independent variables in the mixed models were group (a binary variable equal to 1 for pulmonary rehabilitation group and 0 for the usual care group) and time point (a categorical variable equal to discharge for the 6-week assessment and baseline for the baseline assessment), and group × time point interaction terms were included in the models. A random intercept term was included for each subject. For continuous outcomes, the lmer function from the lme4 library^65^ was used. Estimated marginal means for continuous outcome were calculated using the ggpredict function from the ggeffects library.^66^ Adjusted mean differences for continuous outcomes along with 95% confidence intervals (CIs) and *P* values were generated using the difflsmeans function from the lmerTest library.^67^ The GLMM framework was chosen as both continuous and ordinal dependent variables can be modeled, the GLMM can handle missing data in a way that reduces bias and potential loss of statistical power (ie, GLMM does not rely on listwise deletion), and the GLMM can accommodate subjects with differing numbers of repeated measures. Statistical significance was set at *P* <.05. The number (%) achieving the chosen MCIDs of outcomes are also presented.

## Results

### Participants’ flow through the trial

**Figure 1:**
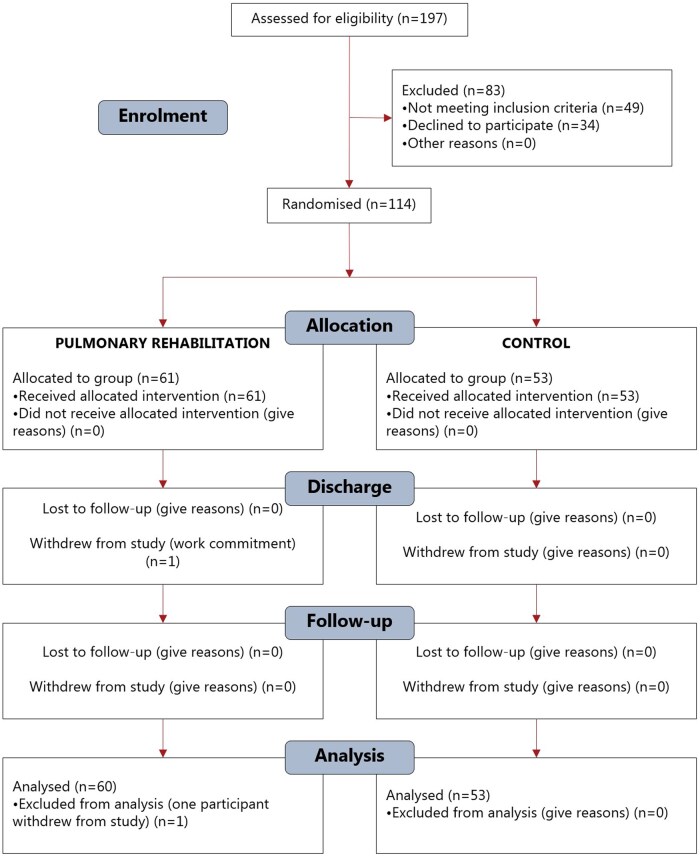
Consolidated Standards of Reporting Trials (CONSORT) diagram for the study flow.

A total of 197 patients with PTLD were screened for the study between March 4, 2021 and June 14, 2022, of whom 163 (82.7%) expressed interest after a brief description of the study. One hundred forty-eight patients (75.1%) were eligible to participate (Figure 1). Of the 148 eligible patients, 114 (77.0%; 57.9% of screened) agreed to participate in the study. Reasons for declining participation (*n* = 34 [23.0%]) were mainly due to family commitments (*n* = 14 [41%]), work commitments (*n* = 10 [29.4%]), studying/education commitments (*n* = 7 [20.5%]), and a lack of available time (*n* = 3 [9.1%]). Of the 61 participants randomized to the intervention arm, 58 participants (95.2%) attended 12 pulmonary rehabilitation sessions and 60 participants (98.4%) attended 75% (9/12) of pulmonary rehabilitation sessions (Supplementary Material A). One participant in the intervention group did not complete the study, withdrawing after attending 6 pulmonary rehabilitation sessions due to work commitments. Face-to-face discharge assessments (6 weeks post-baseline) and telephone follow-up assessments were conducted for all remaining participants (*n* = 113; intervention group *n* = 60 and control group *n* = 53). Full baseline characteristics are provided in Table 1.

**Table 1:** Baseline characteristics.

Baseline characteristics	**Whole sample (*N*** = **114)**	**Intervention (*n*** = **61)**	**Control (*n*** = **53)**
**Age, y, mean (SD)**	44.5 (15.6)	44.4 (14.8)	44.7 (16.5)
**Sex, No. (%)**			
** Male**	59 (51.8)	27 (44.3)	32 (60.4)
** Female**	55 (48.2)	34 (55.7)	21 (39.6)
**Post-BD FEV_1_, L, mean (SD)** ^a^	2.59 (0.96)	2.38 (0.89)	2.84 (0.98)
**Post-BD FVC, L, mean (SD)** ^a^	3.44 (1.00)	3.18 (0.87)	3.75 (1.06)
**Post-BD FEV_1_/FVC ratio, mean (SD)** ^a^	0.75 (0.16)	0.74 (0.18)	0.75 (0.13)
**Smoking status, No. (%)**			
** Never**	58 (50.9)	33 (54.1)	25 (47.2)
** Current**	29 (25.4)	12 (19.7)	17 (32.1)
** Former**	27 (23.7)	16 (26.2)	11 (20.8)
** Pack-years, median [IQR]^b^**	4.3 [17.3]	2.8 [17.7]	5.0 [16.8]
**Biomass daily exposure, No. (%)**			
** Yes**	72 (63.2)	40 (65.6)	32 (60.4)
** No**	42 (36.8)	21 (34.4)	21 (39.6)
**Biomass years exposed, median [IQR]**	10.0 [25.0]	10.0 [19.0]	10.5 [25.0]
**Times treated for TB, median [IQR]**	1 [0]	1 [0]	1 [0]
**Times treated for TB, No. (%)**			
** 0**	1 (0.9)	1 (1.6)	0 (0)
** 1**	93 (81.6)	49 (80.3)	44 (83.0)
** 2**	14 (12.3)	8 (13.1)	6 (11.3)
** ≥3**	6 (5.2)	3 (4.9)	3 (5.7)
**Abnormal chest X-ray, No. (%)**	79 (69.3)	41 (67.2)	38 (71.7)
** Fibrosis**	66 (57.9)	36 (59.0)	30 (56.6)
** Nodules**	4 (3.5)	3 (4.9)	1 (1.9)
** Infiltrates**	2 (1.8)	0 (0)	2 (3.8)
** Pleural effusion**	4 (3.5)	1 (1.6)	3 (5.7)
** Masses (≥30 mm diameter)**	3 (2.6)	1 (1.6)	2 (3.8)
**Respiratory-related treatments, No. (%)**			
** ICS**	2 (1.8)	1 (1.6)	1 (1.9)
** LABA**	2 (1.8)	2 (3.3)	0 (0)
** LAMA**	3 (2.6)	2 (3.3)	1 (1.9)
** ICS/LABA**	1 (0.9)	0 (0)	1 (1.9)
** SABA**	4 (3.5)	3 (4.9)	1 (1.9)
** SAMA**	5 (4.4)	2 (3.3)	3 (5.7)
** Antihistamines**	1 (0.9)	1 (1.6)	0 (0)
** Cough syrup**	6 (5.3)	2 (3.3)	4 (7.5)
** Mucolytics**	4 (3.5)	1 (1.6)	3 (5.7)
** Antibiotics**	2 (1.8)	1 (1.6)	1 (1.9)
**HIV status, No. (%)**			
** Positive**	0 (0)	0 (0)	0 (0)
** Negative**	57 (50.0)	34 (55.7)	23 (43.4)
** Unknown**	57 (50.0)	27 (44.3)	30 (56.6)
**Comorbidities, No. (%)**			
** Cardiac disease**	23 (20.2)	14 (23.0)	9 (17.0)
** Peripheral vascular disease**	19 (16.7)	8 (13.1)	11 (20.8)
** Hypertension**	23 (20.2)	11 (18.0)	12 (22.6)
** Diabetes**	9 (7.9)	5 (8.2)	4 (7.5)
** Kidney disease**	19 (16.7)	11 (18)	8 (15.1)
** Arthritis**	25 (21.9)	11 (18)	14 (26.4)
** Mental health disorder**	2 (1.8)	0	2 (3.8)
**Hospitalizations in last 12 mo, median [IQR]**	0 [0]	0 [0]	0 [0]
**Hospitalizations in last 12 mo, No. (%)**			
** 0**	100 (87.7)	53 (86.9)	47 (88.7)
** 1**	12 (10.5)	7 (11.5)	5 (9.4)
** 2**	1 (0.9)	0 (0)	1 (1.9)
**Employment status, No. (%)**			
** In paid work (employed)**	35 (30.7)	16 (26.2)	19 (35.8)
** In paid work (self-employed)**	10 (8.8)	5 (9.4)	5 (8.2)
** In unpaid work**	3 (2.6)	2 (3.3)	1 (1.9)
** Not in work**	66 (57.9)	38 (62.3)	28 (52.8)
**Household income (som/month), No. (%)**			
** ≤5700**	11 (9.6)	6 (9.8)	5 (9.4)
** 5701-12** **500**	29 (25.4)	18 (29.5)	11 (20.8)
** 12** **501-19** **500**	21 (18.4)	10 (16.4)	11 (20.8)
** 19** **501-26** **000**	22 (19.3)	10 (16.4)	12 (22.6)
** 26** **001-33** **000**	19 (16.7)	9 (14.8)	10 (18.9)
** >33** **000**	12 (10.6)	8 (13.0)	4 (7.5)
**Education level, No. (%)**			
** <9 y of schooling**	3 (2.6)	2 (3.3)	1 (1.9)
** School (9 years)**	2 (1.8)	2 (3.3)	0
** School (11 years)**	45 (39.5)	22 (36.1)	23 (43.4)
** College**	26 (22.8)	17 (27.9)	9 (17.0)
** University/Academy**	38 (33.3)	18 (29.5)	20 (37.7)

**Abbreviations:** BD, bronchodilator; FEV1, forced expiratory volume in 1 second; FVC, forced vital capacity; HIV, human immunodeficiency virus; ICS, inhaled corticosteroid; IQR, interquartile range; LABA, long-acting β-agonist; LAMA, long-acting muscarinic antagonist; SABA, short-acting β-agonist; SAMA, short-acting muscarinic antagonist; SD, standard deviation; TB, tuberculosis.

a Spirometry: *n* = 102 (*n* = 56 intervention; *n* = 46 control).

b Pack-years: *n* = 56 (*n* = 28 intervention; *n* = 28 control).

### Intention-to-treat analysis

#### Primary outcome: ISWT

Compared with the control group, change from baseline in ISWT distance was significantly greater in the intervention group (Table 2). A summary of the mixed model is given in Table S1a of the Statistical Supplement. Compared with the control group, change in ISWT distance from baseline was significantly greater in the intervention group (123.0 m [95% CI, 81.1-164.8 m], *P* <.001). The mean change in the intervention group exceeded the MCID of 35 m (119.3 m [95% CI, 90.7-147.9 m]; 81% meeting MCID) and that in the control group did not (−3.7 m [95% CI, −34.4 to 27.1 m]; 17% meeting MCID) (Table S1b).

**Table 2:** Changes in primary and secondary outcomes between baseline and discharge visits (discharge − baseline).

Outcome variables	Intervention (*n* = 60)				Control (*n* = 53)				Difference in change between groups	*P* value	MCID/MID
	Pre	Post	Mean change (95% CI)	*P* value (time)	Pre	Post	Mean change (95% CI)	*P* value (time)			
**Primary outcome**											
** ISWT, m**	444.3 (405.0-483.5)	563.6 (524.1-603.0)	119.3 (90.7-147.9)	<.0001	474.2 (431.7-516.8)	470.6 (428.0-513.1)	−3.7 (−34.4 to 27.1)	.814	123.0 (81.1-164.8)	<.001	35
**Secondary outcomes**											
** MRC score^a^**	2.7 (2.5-2.9)	1.5 (1.4-1.7)	−1.2 (−1.3 to −1.0)	<.0001	2.6 (2.4-2.8)	2.6 (2.4-2.8)	−0.04 (−0.2 to 0.1)	.659	−1.1 (−1.4 to −0.9)	<.001	1
** CCQ score (total)**	2.3 (2.1-2.5)	0.9 (0.7-1.1)	−1.3 (−1.5 to −1.2)	<.0001	2.1 (1.9-2.4)	2.3 (2.1-2.5)	0.1 (−0.1 to 0.3)	.194	−1.4 (−1.78 to −1.2)	<.001	0.4
** CAT score**	14.3 (12.8-15.8)	6.6 (5.1-8.0)	−7.7 (−9.0 to −6.5)	<.0001	14.1 (12.6-15.7)	15.1 (13.5-16.7)	1.0 (−0.4 to 2.3)	.149	−8.7 (−10.5 to −6.9)	<.001	2.0
** HADS score (depression)**	6.0 (5.2-6.9)	2.5 (1.7-3.3)	−3.6 (−4.3 to −2.8)	<.0001	6.7 (5.8-7.6)	7.4 (6.5-8.3)	0.7 (−0.1 to 1.5)	.076	−4.3 (−5.4 to −3.2)	<.001	2.0
** HADS score (anxiety)**	7.3 (6.5-8.0)	2.8 (2.1-3.6)	−4.5 (−5.2 to −3.7)	<.0001	7.3 (6.5-8.1)	8.2 (7.4-9.0)	0.9 (0.1-1.7)	.020	−5.4 (−6.4 to −4.3)	<.001	2.0
** EQ-5D-5L VAS**	73.0 (69.7-76.2)	90.6 (87.3-93.8)	17.6 (14.4-20.8)	<.0001	72.8 (69.4-76.3)	70.3 (66.8-73.7)	−2.5 (−6.0 to 0.9)	.144	20.2 (15.5-24.9)	<.001	7.0
** Sit-to-stand, sec^b^**	2.11 (2.04-2.19)	1.95 (1.88-2.03)	−0.16 (−0.20 to −0.12)	<.0001	2.12 (2.04-2.20)	2.18 (2.10-2.26)	0.06 (0.01-0.10)	.012	−0.22 (−0.28 to −0.16)	<.001	1.7
** ESWT, sec^b^ ^,^ ^c^**	6.15 (6.04-6.25)	6.37 (6.27-6.47)	0.22 (0.13-0.31)	<.0001	6.21 (6.10-6.32)	6.08 (5.97-6.19)	−0.14 (−0.23 to −0.04)	.006	0.36 (0.23-0.49)	<.001	147–279

For continuous variables, pre and post values are predicted values of outcome from the generalized linear mixed model (GLMM), and mean change is adjusted mean difference from GLMM. For ordinal variables (MRC score), pre and post values are predicted probability of response level from GLMM, and mean change is adjusted mean difference in predicted probability from GLMM.

Abbreviations: CAT, COPD Assessment Test; CCQ, Clinical COPD Questionnaire; CI, confidence interval; COPD, chronic obstructive pulmonary disease; ESWT, endurance shuttle walking test; HADS, Hospital Anxiety and Depression Scale; ISWT, incremental shuttle walking test; MCID, minimal clinically important difference; MID, minimal important difference; MRC, Medical Research Council; VAS, Visual Analogue Scale.

a Mixed model, MRC as continuous variable.

b Log transformed.

c *n* = 59 for intervention group.

#### Secondary outcomes

Secondary outcome ITT analyses and outputs are provided in Table 2.

##### Health-related quality of life

Compared with the control group, change from baseline in CAT was significantly greater in the intervention group (−8.7 [95% CI −10.4 to −6.9], *P* <.0001; Table S2a). Change in CAT score for the intervention group exceeded the MCID of 2 (−7.6 [95% CI, −9.0 to −6.2]; 78% met MCID) (Table S2b).

Compared with the control group, change from baseline in CCQ total score was significantly greater in the intervention group (−1.4 [95% CI, −1.7 to –1.9], *P* <.0001; Table S3a). Change in CCQ total score for the intervention group exceeded the MCID of 0.4 (−1.3 [95% CI, −1.5 to −1.1]; 85% met MCID) (Table S3b).

Compared with the control group, change from baseline in EQ-5D-5L VAS was significantly greater in the intervention group (20.2 [95% CI, 15.5-24.9], *P* <.0001; Table S4a). Change in EQ-5D-5L VAS for the intervention group exceeded the MCID of 7 (17.9 [95% CI, 14.4-21.3]; 77% met MCID) (Table S4b).

##### Anxiety and depression

Compared with the control group, change from baseline in HADS-Anxiety was significantly greater in the intervention group (−5.4 [95% CI, −6.4 to −4.3], *P* <.0001; Table S5a). Compared with the control group, change from baseline in HADS-Depression was significantly greater in the intervention group (−4.3 [95% CI, −5.3 to −3.2], *P* <.0001; Table S6a). Changes exceeded the MCID of 2 for the intervention group for in HADS-Anxiety (78% met MCID; Table S5b) and HADS-Depression (70% met MCID; Table S6b).

##### Physical measures

Compared with the control group, change from baseline in 5STS was significantly greater in the intervention group (−0.22 [95% CI, −0.28 to −0.16], *P* <.0001; Table S7a). Compared with the control group, change from baseline in ESWT was significantly greater in the intervention group (0.36 [95% CI, 0.23-0.49], *P* <.001; Table S8a). The mean change in the intervention group did not exceed the MCID for 5STS (35% met MCID; Table S7b) or ESWT (30% met the MCID; Table S8b).

### Per protocol analysis

Per protocol results were comparable to findings from the ITT (Statistical Supplement, Tables S9a to S16b). Per protocol outputs are summarized in Table S17.

### 12-week outcomes

When followed up by telephone at 12 weeks post-baseline (6 weeks after intervention completion), difference in change between groups observed between baseline and discharge was maintained between discharge and follow-up (Table S18). Per protocol results were comparable to findings from the ITT (Table S19).

### Health economic analysis

Outcomes for the EQ-5D-5L questionnaire are reported in Table 3. The intervention group saw a significant increase in QALYs over the control group (0.2 [95% CI, 0.1-0.2]). The estimate of incremental QALY change for the intervention group over the control group is combined with information reflecting the cost per patient of delivering the intervention, to calculate the program cost per QALY gained. We calculate a total program cost of US$5686.5, or an average cost of US$95 for every patient who received the intervention. Details of these calculations are reported in Supplementary Material B. This gives a cost of US$614.1 per QALY (95% CI, $464.7-$763.5). After adjusting for purchasing power, program cost is US$2143.2 per QALY (95% CI, $1621.9-$2663.9).

**Table 3:** Health economic outcomes: EQ-5D-5L, QALYs, and cost effectiveness of pulmonary rehabilitation.

Outcome	Change in outcome between assessment and discharge		(B) − (A) Difference	95% CI for difference
	(A) Control	(B) Intervention		
**Mobility problems**	0.2 (0.1)	−0.6 (0.1)	−0.8 (0.1)	−1.0 to 0.5
**Self-care problems**	0.0 (0.1)	−0.6 (0.1)	−0.6 (0.1)	−0.9 to −0.4
**Problems with usual activities**	0.2 (0.1)	−0.6 (0.1)	−0.8 (0.1)	−1.0 to −0.5
**Pain and discomfort**	0.0 (0.1)	−0.7 (0.1)	−0.8 (0.1)	−1.0 to −0.6
**Anxiety and depression**	0.1 (0.1)	−0.8 (0.1)	−0.9 (0.1)	−1.1 to 0.6
**Overall health today**	−2.5 (1.7)	17.6 (1.7)	20.1 (2.4)	15.4-24.9
**QALYs**	−0.03 (0.01)	0.13 (0.01)	0.15 (0.02)	0.12-0.19
**Cost per QALY (US$)**	…	…	614.1	464.7-763.5
**Cost per QALY adjusted for purchasing power (US$)**	…	…	2143.2	1621.9-2663.9

Abbreviations: CI, confidence interval; QALY, quality-adjusted life-year.

Regarding the cost-effectiveness component of these analyses, the manuscript was checked against the CHEERS 2022 reporting checklist, which is provided in the Supplementary Material.

### Safety

Adverse events were reported in 3 (2.6%) participants, of whom 2 (3.3%) were in the intervention group and 1 (1.9%) was in the control group. The adverse events were exacerbations of their condition (*n* = 1; intervention, unrelated to study processes), leg pain (*n* = 1; intervention related), and back strain (*n* = 1; control, unrelated to study processes).

### Protocol deviations

To minimize risk of infection during the COVID-19 pandemic, follow-up measures (12 weeks post-baseline) were conducted by telephone only. Therefore, only patient-reported outcomes are available at this timepoint.

Due to unexpected data loss, we were unable to examine physical activity as a secondary outcome of the trial.

## Discussion

In this first fully powered randomized controlled trial, we have shown clinical and cost effectiveness of culturally adapted supervised pulmonary rehabilitation compared to usual care for people living with PTLD. Improvements in the primary outcome of maximal exercise capacity exceeded the available MCID following pulmonary rehabilitation.^43^ Secondary outcomes, including health-related quality of life and symptom burden, showed significant improvements in the pulmonary rehabilitation group. We deliberately developed and tested a culturally adapted pulmonary rehabilitation intervention, recognizing the need to ensure local relevance and appeal to patients and other key stakeholders.^34^ The value of this approach was evidenced by the high trial uptake and completion of the intervention group. Collectively our data provide strong evidence for the benefit of culturally adapted hospital-based pulmonary rehabilitation for people living with PTLD, supporting the WHO Rehabilitation 2030 initiative to globally scale up rehabilitation interventions and provide robust evidence for pulmonary rehabilitation in an underrepresented population.^68^

Our primary outcome of maximal exercise capacity (ISWT distance) improved in the pulmonary rehabilitation group, with a magnitude of change well beyond the conventional MCID of 35 m,^43^ sustained at 6 weeks post-intervention. Improvements across a range of self-reported health-related quality of life outcomes reflected changes in physical outcome measures, caveated on these outcomes not being powered. Observed improvements are supported by the previously limited mixed quality evidence available, summarized in a systematic review of interventions for preventing post-TB sequalae.^33^ Findings align with international data in chronic obstructive pulmonary disease (COPD), including latest UK audit findings from the National Respiratory Audit Programme showing that 59% and 71% of pulmonary rehabilitation completers with COPD achieve clinically meaningful improvements in exercise capacity and health status, respectively.^69^

Our study was the first to examine the cost effectiveness of pulmonary rehabilitation in the context of LMICs and PTLD, demonstrating a highly cost-effective intervention, in keeping with National Institute for Health and Care Excellence (NICE) guidelines from the United Kingdom.^70^

The resulting cost per QALY is 27% of Kyrgyzstan’s 2023 per capita GDP, below the WHO’s LMIC cost-effectiveness threshold of 1 to 3 times GDP, and within more conservative threshold estimates for Kyrgyzstan.^71^ Our findings evidence the potential for pulmonary rehabilitation to be sustainably embedded in health systems in resource-limited settings, such as Kyrgyzstan. Pulmonary rehabilitation has previously been shown to be a cost-effective intervention for patients living with chronic lung diseases, including COPD and interstitial lung disease, in high-income countries.^72^^,^^73^

Overall, evidence generated in our study supports the WHO Rehabilitation 2030 initiative^68^ to scale up rehabilitation worldwide and to bolster research efforts, policy, and funding provisions for the neglected condition of PTLD.^15^ The present study expands the availability of robust evidence for rehabilitation in typically underrepresented populations, such as individuals living with PTLD and in many LMICs where demand for pulmonary rehabilitation greatly outweighs current capacity.^74^

Due to the nature of pulmonary rehabilitation interventions, it was not possible to blind patients to their allocation. Participants were informed of their allocation following completion of their baseline visit. Although similar between groups, only a small proportion of participants were on prescribed medication prior to pulmonary rehabilitation. This was not altered during the study period and may have blunted responses to the intervention, leading to underestimations of its effect. There are a lack of Central Asian and PTLD-specific outcome measures and MCIDs, which may not accurately reflect our younger population compared with other CRDs such as COPD, for which the majority of tools are developed. However, the magnitude of improvements observed in our study greatly exceeded conventional MCIDs across a wide range of outcomes, thus unlikely to influence the overall findings of the trial. A limitation to the cost-effectiveness analysis is that there are currently no available EQ-5D-5L value sets specific to Kyrgyzstan for calculating QALYs. To account for this, we used all available country-specific value sets in our sensitivity analysis and provided the most conservative estimate. However, we cannot rule out that Kyrgyzstan-specific value sets will yield a much lower estimated QALY increase.

A longer-term follow-up would have provided greater insights into the maintenance of health-enhancing behaviors and longevity of the observed clinical benefits. This was a single-center randomized controlled trial with participants recruited primarily from the Bishkek region of Kyrgyzstan. Given the varied topography of the country, including mountainous high-altitude areas, modifications may be necessary to optimize pulmonary rehabilitation delivery. Our approach of adapting guideline-based pulmonary rehabilitation may be transferred to other settings, including existing pulmonary rehabilitation services where challenges with referral, uptake, and retention remain.

Our study demonstrated the clinical and cost effectiveness of culturally adapted pulmonary rehabilitation for adults living with PTLD. Further work is needed to explore how to scale up the availability of pulmonary rehabilitation within healthcare systems globally, particularly in LMICs where services are sparsely available but the burden of disability from chronic respiratory disease is substantial.

## Supplementary Material

aaoag062_Supplementary_Data

## Data Availability

The data underlying this article are not publicly available but may be shared upon reasonable request. Expressions of interest in collaboration, contributing research data to the database, or accessing existing data should be directed to the RECHARGE Scientific Committee at recharge@le.ac.uk.
